# Supplementary material for: Frailty among Older People during the First Wave of the COVID-19 Pandemic in The Netherlands
Source: Int J Environ Res Public Health. 2022 Mar 19;19(6):3669. doi: 10.3390/ijerph19063669 (PMC8950938; doi:10.3390/ijerph19063669)
Supplement: Supplementary file 1 [file ijerph-19-03669-s001.zip › ijerph-1605182-supplementary.pdf]

Table S1. Items and scores of the GFI in the Lifelines Covid-19 questionnaire

Item no.	GFI-items	GFI-items in Lifelines Covid-19 questionnaire
1	Can you independently perform the following activities without any help from someone else, possibly with the help of a cane, walker or wheelchair?<br>Get groceries and run errands<br>*Yes = 0; No = 1*	Can you independently perform the following activities without any help from someone else, possibly with the help of a cane, walker or wheelchair?<br>Get groceries and run errands<br>*Yes = 0; No = 1*
2	Can you independently perform the following activities without any help from someone else, possibly with the help of a cane, walker or wheelchair?<br>Get dressed/undressed<br>*Yes = 0; No = 1*	Can you independently perform the following activities without any help from someone else, possibly with the help of a cane, walker or wheelchair?<br>Get dressed/undressed<br>*Yes = 0; No = 1*
3	Can you independently perform the following activities without any help from someone else, possibly with the help of a cane, walker or wheelchair?<br>Move outdoors (around house, to neighbors)<br>*Yes = 0; No = 1*	Can you independently perform the following activities without any help from someone else, possibly with the help of a cane, walker or wheelchair?<br>Move outdoors (around house, to neighbors)<br>*Yes = 0; No = 1*
4	Can you independently perform the following activities without any help from someone else, possibly with the help of a cane, walker or wheelchair?<br>Go the toilet<br>*Yes = 0; No = 1*	Can you independently perform the following activities without any help from someone else, possibly with the help of a cane, walker or wheelchair?<br>Go the toilet<br>*Yes = 0; No = 1*
5	What score would give your physical fitness (from 0 to 10)<br>*0-6=1; 7-10 = 0*	What score would give your physical fitness (from 0 to 10)<br>*0-6=1; 7-10 = 0*
6	Do you have problems in every day life due to poor vision?<br>*Yes = 1; No = 0*	Do you have problems in every day life due to poor vision?<br>*Yes = 1; No= 0*
7	Do you have problems in every day life due to poor hearing?<br>*Yes= 1; No = 0*	Do you have problems in every day life due to poor hearing?<br>*Yes = 1; No = 0*
8	Have you lost a lot of weight in the past period without wanting to (6 kg in 6 months or 3 kg in one month)?<br>*Yes = 1; No = 0*	Have you lost a lot of weight in the past period without wanting to (6 kg in 6 months or 3 kg in one month)?<br>*Yes = 1; No = 0*
9	Do you take 4 or more different types of medicine?<br>*Yes = 1; No = 0*	Number of different medicines from the medication checklist.<br>*≥4 = 1; <4 = 0*
10	Do you have memory problems?<br>*Yes = 1; No/sometimes = 0*	Do you have memory problems?<br>*Yes = 1; No/sometimes = 0*
11	Do you ever experience emptiness around you?<br>*Sometimes/yes = 1; No = 0*	How often do you feel disconnected from others in the last 7 days?<br>*Sometimes/often = 1; Almost never/never = 0*
12	Do you ever miss people around you?<br>*Sometimes/yes = 1; No = 0*	How often did you feel alone in the past 7 days?<br>*Sometimes/often = 1; Almost never/never = 0*
13	Do you ever feel let down?<br>*Sometimes/yes = 1; No = 0*	How often did you feel let down in the past 7 days?<br>*Sometimes/often = 1; Almost never/never = 0*

Online Supplement 1. continued

Item no.	GFI-items	GFI-items in Lifelines Covid-19 questionnaire
14	Have you felt gloomy or depressed recently?<br>*Sometimes/yes = 1; No = 0*	During the past seven days, have you felt constantly depressed or depressed for most of the day, almost every day?<br>*Yes = 1; No = 0*
15	Have you felt nervous or afraid recently?<br>*Sometimes/yes = 1; No = 0*	a. In the past seven days, have you often felt restless, loaded or nervous?<br>*Yes = 1; No = 0*<br>b. In the past seven days, have you been worrying excessively and worried about multiple daily life problems of work, of home or about your immediate environment?<br>*Yes = 1; No = 0*<br>*Both items yes = yes; 1 item yes = sometimes; 2 items no = no*<br>*Sometimes/yes = 1; No = 0*

Items not shaded gray are identical to the original GFI in the Lifelines Covid-19 questionnaire.
Items shaded gray are not identical to the original GFI in the Lifelines Covid-19 questionnaire.
